# Supplementary material for: Temporal regularities of vocal exchange in Java sparrows
Source: PLoS One. 2025 Jul 23;20(7):e0327024. doi: 10.1371/journal.pone.0327024 (PMC12286380; doi:10.1371/journal.pone.0327024)
Supplement: Attachment — Submitted filename: Reply_to_reviewer.pdf [file pone.0327024.s002.pdf]

Reply to reviewers and editors

We sincerely appreciate your evaluation and the insightful reviews of our manuscript submitted to *PLoS ONE*. We are particularly grateful for your recognition of our efforts in clarifying the temporal patterns of vocal exchange in Java sparrows. We also appreciate your positive feedback regarding the clarity of our experimental methods and analyses.

As turn-taking and vocal exchange are fundamental features of mammal communication, including human conversation, it has been assumed in previous research that similar patterns would be observed in birds as well. However, our study, which demonstrates that such patterns are not necessarily present in Java sparrows, provides valuable insight into this area. We believe our findings add significant value to the existing body of research.

That said, as pointed out by the editor and two reviewers, our original manuscript overgeneralized the findings from a single sparrow species to "avian" characteristics. Based on the valuable comments we received, we have made substantial revisions to the manuscript. We believe that, with these extensive revisions, the manuscript is now well-suited for publication in *PLoS ONE*.

Below, we provide our detailed responses to each comment received. The numbers assigned to the reviewer comments (e.g., R1-1) correspond to the highlighted sections in the marked-up copy of the revised manuscript.

We sincerely hope to address the concerns you raised regarding the previous version of the manuscript.

Hiroki Koda, Noriko Kondo, Sota Kikuchi

Reviewer #1:

R1-1

Kikuchi et al. describe an experiment in which they examine call latency within and between Java sparrows. They claim that repeated call latency (when a bird continues calling without a response from another present bird) is shorter than similar types of intervals observed in mammals and other birds. While the use of comparative metrics is valuable, it's unclear the extent to which these data are appropriate for their question and subsequently whether their conclusions are appropriate.

I appreciate the evaluation of how social dynamics within groups of species informs their question, but the broad generalization of the 'fundamental difference of social structure in mammals and birds" (line 124) is quite sweeping and not testable using a single species of bird. While they compare their findings to published zebra finch data, it seems as though they're own analyses differ significantly from those previous studies, so it is difficult to draw conclusions as to any similarities or differences between the species.

You are absolutely correct. It was inappropriate to generalize our findings to all avian species based solely on a single species, the Java sparrow. In response, we have revised the relevant sections, including the title, to mitigate the generalization and refrain from making conclusions that apply to all avian species. The specific revisions are as follows:

> **Title:** Temporal regularities of vocal exchange in Java sparrows
>
> **L. 114-120**
> Regarding the need to maintain group cohesiveness, mammals and birds are expected to differ from other individuals in their anticipated responses. Specifically, mammals are more likely to expect and anticipate responses, whereas birds may not share the same expectations. However, research examining the temporal characteristics of exchanges and repeated transitions in this field has been limited to zebra finches. Therefore, to conduct a comprehensive comparison of vocal turn-taking between these two taxa, it is necessary to expand our knowledge of vocal turn-taking in birds by investigating species other than zebra finches.

R1-2

Also, it unclear as to what question(s) they're attempting to test. Is it the calling pattern of single individual when in the presence/absence of another bird, or whether these calling patterns are fundamentally different from other bird/mammalian species? The authors should more clearly clarify the goal of their study. And, if they're attempting to both questions, it's not clear that they have sufficient evidence for the latter.

My main concern with the manuscript is that I'm uncertain as to what the researchers were measuring (all call intervals regardless of social context or only call intervals when in the presence of a conspecific (F2F). Several sentences are difficult to interpret and could be clarified – while these are just a few examples, there are others that may contribute to difficulty in understanding the mechanics of the manuscripts.

- *Line 155: "They could usually hear and see each other"*
- *Line 160: "We designed the experimental space in which individual cages could be placed three-dimensionally"*
- *Line 189: "faced over the cage"*

Our goal was to determine whether the general trends observed in previous studies on turn-taking in mammals, as shown in Fig. 1, could also be observed in Java sparrows. The answer to your question, "*Is it the calling pattern of a single individual when in the presence/absence of another bird, or whether these calling patterns are fundamentally different from other bird/mammalian species?*" would be the latter.

The reason why our research objectives, results, and discussion may not have been properly understood is related to the next comment—namely, that our analysis target was not sufficiently described. We kindly ask you to consider the following comment in conjunction with this one.

We are confident that, after doing so, you will recognize that the experimental results in this study on Java sparrows differ from the turn-taking characteristics illustrated in Fig. 1.

Regarding the points you raised as examples of ambiguity in our experiment, we have made the following revisions in the revised version.

L.142-143
All individuals were housed separately in identical cages (38 cm high × 31 cm wide × 25 cm deep) within the same housing area, where they could hear and see each other.

L.151-153
Within the chamber, we designed an experimental space that allowed individual cages to be placed opposite to each other, facing one another (Fig. 2A).

L.178-179
In the F2F phase, the other individual (facing individual) was placed opposite the subject bird facing it.

R1-3

My understanding of the methodology is that Java sparrows called in two social contexts: alone (PRE and POST) and F2F. Total call number was greater in the F2F condition compared to the PRE/POST conditions. In addition, they measured the call interval in each of these contexts. In the F2F condition, the call interval was split into either an 'exchange transition' (a response to a partner bird) or a 'repeated transition' (the interval between two consecutive calls from the same individual).

By definition, 'exchange transitions' (te) can only occur in the F2F condition. However, 'repeated transitions' (tr) can occur in any social context. It appears the tr have all been grouped together. It would be helpful if the authors included an analysis of solely the F2F (not including any data from the PRE/POST conditions) phase. If that is what the authors did, it should be made clearer that only call intervals from the F2F phase were evaluated. Certainly, the birds must use visual and auditory cues to determine the presence/absence of another bird – the low tr interval could be explained by birds repeatedly calling in the absence of another bird in the PRE/POST conditions – the birds would not be expecting a response, so have no reason to avoid calling in a typical overlapping time interval. It would also be helpful to quantify the total percentage of returned calls – is the rate of return similar to what is observed in stranger zebra finches (Benichov et al., 2017)?

We apologize for the insufficient description of our methods and the structure of the manuscript, which may have led to a miscommunication of our experimental details. In the revised manuscript, we have made several modifications to ensure that our experimental procedures are clearly conveyed. The 'Statistical evaluation of the number of calls across experimental phases' section was initially placed at the beginning of the Statistics section in the original manuscript. This arrangement could have led to a misunderstanding, as it might have appeared that we were comparing call intervals between the three phases. However, the comparison between the three phases was conducted to examine whether the call frequency in Java sparrows was influenced by other individuals. Therefore, in the revised manuscript, we have moved this section to the end of the Statistics section (L.286-297 in the revised manuscript).

Additionally, we have added clarifications to ensure the purpose of this analysis is clear. Furthermore, by appropriately placing the term ‘F2F phases’ in the manuscript, we have made the content of the analysis clearer. Specifically, the following changes have been made:

> **L.230**
> For the analysis of vocal exchange, we only used recordings of the F2F phases.

> **L.241-243**
> To investigate whether the temporal structure of the exchange transitions and repeated transitions in F2F phases differed statistically, we applied Gaussian mixture models (GMMs) to the time interval data of each transition.

A comparison of tr in PRE/F2F/POST conditions would strengthen the manuscript to consider the effect of social context. Similarly, if the authors have video recordings of the birds, an account of whether the ‘partner’ bird is socially engaging the with the test bird, and thus, potentially more likely to respond to the test bird would also be a valuable addition. Lines 354-356 state that it’s the pseudo turn-taking data that lead to the strong suggestion that the short component identified in the exchange transition originated from the response call – the actual data from the two birds should be what leads to this suggestion because those data should and do exist.

In this experiment, we focused exclusively on the F2F phase, as our interest was in vocal exchanges between two birds, and did not compare it to the PRE and POST phases. We appreciate your valuable comments on videos. However, unfortunately, we do not have any videos available for analysis in this study. However, we sincerely appreciate your comment and will ensure to collect videos for more detailed analysis in future experiments.

R1-4

I admit that I am not super familiar with the intricacies of GLMMs and GMMs. If the the GLMM considers the social context (alone vs. conspecific present), it would be helpful to more clearly illustrate and describe that process, along with various acronyms (e.g. AIC) that are used.

In the revised manuscript, we have clarified that a comparison of the three phases was conducted in the GLMM description. As per your suggestion, we have also spelled out AIC instead of using the abbreviation.

> **L.293-295**
> By comparing the Akaike information criterion (AIC), we estimated which model outperformed the null model in which the condition was removed from the fixed effects.

R1-5

In the abstract, the authors state that the te latency is ~170ms. Nowhere in the manuscript do the authors report this result or explain its relevance (or adequately compare it to other

mammalian or avian species). Similarly, they do not report a mean tr latency (for either solo vs. partner contexts) and how it compares to other mammalian or avian species (they report only that it is different – shorter).

As you pointed out, the value of 170 msec was not mentioned in the Results section of the original manuscript. We recognized that this value does not play a significant role in our findings, and as such, we have removed it from both the Abstract and the Discussion. Specifically, the following changes have been made:

> **L.21-22**
> The results revealed that they vocalized at very short intervals following the calls of the other individual.
>
> **L.380-381**
> A comparison of the pseudo turn-taking data and exchange transitions showed a short component only for the exchange transition.

R1-6

Which bird was the female bird? While isolating a single bird's performance in this experiment is likely not fruitful, including the female in the experiment leads to questions of whether the social dynamics between males/males and males/females is different.

We completely agree with your comment that the inclusion of only one female in our experiment could have various potential effects on the results. Considering that our original manuscript lacked sufficient discussion on this matter, we have added the following explanation in the Discussion section of the revised manuscript:

> **L.415-427**
> In our study, Java sparrows were predominantly male, with a single female, and each individual was housed separately. Consequently, there were no individuals with affiliative relationships such as pairs. Given these conditions, it must be acknowledged that the social environment of the birds in our study is a significant simplification of their natural social lives. In zebra finches housed in mixed-sex groups, vocal exchange between pairs has been shown to take priority, with vocal exchanges occurring at shorter intervals between pairs than in non-paired individuals [41]. Additionally, the call interval in mixed-sex groups is shorter than that in groups consisting only of females [59]. Given that, similar to zebra finches, pair bonds are fundamental in Java sparrows, it is reasonable to assume that the temporal regularity of vocal exchange may vary depending on social relationships. Although our study can be regarded as an investigation of the basic temporal regularity of vocal exchange in the absence of pair bonds, future research is essential to examine the influence of sex differences and social relationships on the temporal regularity of vocal exchange.

I recommend several alterations to the figures to also strengthen the data presentation.

We appreciate your comments. We have changed the figures as follows as per your comments. Please note that the figure numbers (Fig 3 and Fig 5) have changed due to the alteration in the order of appearance in the revised manuscript.

R1-7

Figure 1: Include subscripts for the e and r in te and tr. Include a figure caption for 1B and perhaps an explanation for the predicted distributions.

We included subscripts for the te and tr in the Figure1A ("Inter-call interval of exchange transition (te)" and "Inter-call interval of repeated transition (tr)") as has been suggested. We added an explanation of Figure 1B in the caption of Figure 1. We also changed "animal" in the first sentence to "social mammals" to be consistent with the title of Figure 1.

> **L.61-67**
> **Fig 1. Conceptual representation of the mechanism underlying the emergence of temporal regularity in vocal exchange/turn-taking of various social mammals.**
> When social mammals are engaged in a reciprocal vocal exchange (A, top), the individual expects a "response" from the other individual and therefore waits for a response. After the expected response time had elapsed, the individual repeated the vocalization (A, bottom). Fig 1B illustrates the inter-call intervals of vocal exchange in mammals. The inter-call interval of the repeated transitiont_r is longer than that of the exchange transition t_e (A, B).

Figure 2: Figure 2B – include a time scale

We included a time scale bar to Figure 2B.

R1-8

Figure 3: Indicate the female bird in Figure 3A. Figure 3B is unnecessary.

We showed the female bird ID in lowercase letter (b2) in Fig 5 (Fig 3 in the original manuscript). Accordingly, we have changed the caption of Fig 5 as follows.

> **L.350-352**
> **Fig 5. Total number of calls given by six subjects in three experimental phases.**
> (A) The average number of calls in each phase. Each line indicates each bird. The female bird is indicated by the lowercase ID.

We have removed Figure 3B of the original manuscript as per your suggestion.

R1-9

Figure 4-5: These are the critical figures. Figure 4 could be adjusted to account for 'repeated transitions' in the three contexts, to more carefully compare tr vs. te in F2F. (Also, is there a

maximum latency in which a te or tr can occur? If so, what is the justification for those latencies?)

We apologize that we have not clearly explained our experiments in the original manuscript. The Fig 4 shows the comparison of inter-call intervals in only F2F phase. The possible maximum latency of inter-call interval was 900 seconds (the observed longest interval was 763, 883 and 787 seconds for exchange, repeated and pseudo transitions, respectively). The x-axis of the original axis was up to 5 seconds without any justification. Your comment made us realize that there needed to be a justification for the cutoff seconds on the X axis. Therefore, in the current manuscript, we extended the x-axis to 10 seconds as more than 80% of the intervals in each transition were included within 10 seconds. We have added this information to the caption of Fig 4 as follows.

> **L.333-339**
> **Fig 4. Distributions of inter-call intervals of exchange transitions, repeated transitions and pseudo turn-taking data.** The red line represents the exchange transitions, the blue line represents the repeated transitions, and the green line represents the pseudo-turn-taking data. The length of the inter-call interval could be theoretically up to 900 seconds, but the X-axis here is limited to 10 seconds. This is because more than 80% of the inter-call interval was contained within 10 seconds (90.04% of exchange transition, 83.40% of repeated transition and 82.38% of pseudo transition).

Figures 4 and 5 could easily be combined.

Although you have suggested combining Figure4 and 5, we decided to keep them separate. This is because the x-axis in Fig 4 was on a real time scale, while the x-axis in Figure 5 (Fig 3 in the revised manuscript) was log-transformed, and we were concerned that combining figures on different scales would confuse the readers. We believe the reviewer will understand our decision.

R1-10

In Figure 5 (according to the caption), a vertical dotted line represents the estimated values of the mean parameter in the short components). However, Table 5 (and lines 329-330) indicates negative values for those parameter estimates. It is unclear exactly what the dotted lines represent.

We admit that the negative values in Table1 were quite confusing. This is the log-transformed values but it could hinder intuitive understanding. Therefore, we decided to show antilogarithmic values in parentheses in Table1 and changed the caption of Table1 as follows.

> **L.320-323**

Data fitted	Data size (N)	Mean (short, long)	Variance (short, long)	Mixture ratio (short, long)
Exchange transition	1710	-0.728, 0.321<br><br>(0.187, 2.094)	0.023, 0.502<br><br>(1.054, 3.177)	0.322, 0.678
Repeated transition	3350	-0.669, 0.578<br><br>(0.214, 3.784)	0.035, 0.296<br><br>(1.084, 1.978)	0.167,0.833

Table 1 The parameter estimations of the GMMs fitting for the data of exchange transition and repeated transition. The numbers in parentheses in the Mean, and Variance columns represent antilogarithmic values, converted from logarithms back to seconds to help understanding.

Lastly, the title of the manuscript is a bit odd considering that the term 'contagious' is not properly defined nor used until midway through the discussion.

We totally agree with your comment. In the revised manuscript, we have changed the title to 'Temporal regularities of vocal exchange in Java sparrows', which is more aligned with the content of our manuscript.

Overall, as currently written, it is not possible to determine whether the authors conclusions and interpretations are justified due to the description and presentation of the data.

I applaud the authors for utilizing another avian species for a comparative study regarding vocal turn-taking. I also appreciate the pseudo turn-taking data as a control condition, and the inter-rater reliability metrics the authors provided regarding call classification, caller identification, and onset/offset times.

We agree with your comment that our conclusion was overgeneralized, and we have made revisions throughout the manuscript, including the title. We believe these changes have significantly improved the

quality of our manuscript. Additionally, we greatly appreciate your evaluation of our pseudo turn-taking data.

I selected, 'No' in response to 'Is the manuscript presented in an intelligible fashion and written in standard English?'

While the manuscript is written in very readable English, there are enough typographical and language errors that lead to reader confusion that I suggest the authors address in future revisions.

We fully agree with your comment that the English in our original manuscript was not refined. We have thoroughly revised the expressions and addressed this issue by utilizing a language editing service.

Reviewer #2:

Kikuchi et al investigate the temporal patterns of vocal exchanges in Java sparrows, with the aim of comparing communication strategies of birds to those reported in mammals. Specifically, the study aims to tests if, during vocal interactions, Java sparrows wait for a partner's response before they produce their next call (a pattern associated with mammalian vocal turn-taking). The authors recorded face-to-face call interactions in randomized pairs of birds and analyze the temporal patterns of calls. They report that the latencies of call responses do not differ from the intervals between repeated calls of individuals. This is interpreted as evidence that birds do not adjust the timing of their vocalizations by anticipating or waiting for the responsive calls of others. Rather, in contrast to mammals, their call responses are "contagious" or socially triggered by the vocalizations of other individuals. The authors assert that these purely responsive vocal patterns correspond to the relatively fluid composition of avian social groups and aid in synchronizing flock behavior.

The study's methods, analyses, and results are sound and generally presented in a clear, convincing manner. Some of the authors' conclusions, however, extend beyond what was tested with their experimental paradigm, particularly in their generalization across bird species and social contexts. Nevertheless, the work presents valuable data and comparative insights from a species for which little is known regarding interactive vocal behavior. Most concerns described below can be addressed by reducing the generality of some claims and providing additional information, for example regarding the occurrence of overlaps and prevalence of repeated calls.

Comments:

R2-1

1) Lines 20-22, 100-101: The authors state that no study has investigated the intervals between repeated calls of individual birds during interactions. However, intra-individual call patterns have indeed been examined in groups of zebra finches (e.g. Beckers & Gahr 2010 and Stowell et al

2016). The latter study specifically shows that temporal distributions of "self-self" call patterns (i.e. repeated transitions) differ across sexes and call types. And with particular relevance for the current study, patterns of intra-individual call timing were found to differ from responses to others (exchange transitions). "Self-partner" response patterns were found to further differentiate as breeding pairs were established. In addition to identifying excitatory influences of calls from self and others, their analyses also characterized patterns of call suppression, includes strong self-suppression, which would suggest mechanisms more complex than solely excitatory contagion.

We apologize for the oversight in our literature review, where we mistakenly stated that no relevant studies exist. We greatly appreciate you bringing to our attention such valuable references. However, the experiments you mentioned are primarily neurophysiological in nature, and including detailed discussion of them in our manuscript proved somewhat challenging. Therefore, we have cited the study that presents intra-individual call intervals in zebra finches, and have revised our manuscript as follows.

> **L.91-94**
> Although these findings highlight the role of vocal timing adjustments in social interactions, research to date has primarily focused on the intervals between calls produced by different individuals, while studies on the intervals between repeated transitions remain limited (see [41]).

R2-2

Kikuchi et al do acknowledge that in other songbirds, familiarity and pair-bonding can affect the temporal patterns of call responses (e.g. faster, higher-likelihood responses to mates). However, since their experimental design is based on randomized, relatively brief pairings in Java sparrows, and does not take factors such as sex, call type, or affiliative pair bonding into account, the authors should be a bit more conservative with their resulting claims and predictions, rather than generalizing across the diversity of birds and social contexts: "We tentatively predict that avian vocal exchange is established by a mechanism different from that of mammalian vocal exchange in social groups - i.e. birds do not predict and wait in anticipation of vocalizations from other individuals." (Lines 383-386).

We fully agree with your comment that our statement in the original manuscript was overly generalized. We have removed this sentence and revised the preceding statement to specifically focus on Java sparrows, as follows:

> **L.375-377**
> This indicates that, unlike the vocal turn-taking observed in socially communicative mammals, including humans, Java sparrows do not expect or wait for a response from other individuals.

Furthermore, we have added the following paragraph in the Discussion section regarding the inclusion of only one female and the inter-individual relationships:

> **L.415-427**

In our study, Java sparrows were predominantly male, with a single female, and each individual was housed separately. Consequently, there were no individuals with affiliative relationships such as pairs. Given these conditions, it must be acknowledged that the social environment of the birds in our study is a significant simplification of their natural social lives. In zebra finches housed in mixed-sex groups, vocal exchange between pairs has been shown to take priority, with vocal exchanges occurring at shorter intervals between pairs than in non-paired individuals [41]. Additionally, the call interval in mixed-sex groups is shorter than that in groups consisting only of females [59]. Given that, similar to zebra finches, pair bonds are fundamental in Java sparrows, it is reasonable to assume that the temporal regularity of vocal exchange may vary depending on social relationships. Although our study can be regarded as an investigation of the basic temporal regularity of vocal exchange in the absence of pair bonds, future research is essential to examine the influence of sex differences and social relationships on the temporal regularity of vocal exchange.

R2-3

2) The authors state that instances of call overlap are extremely rare (only 2/1712 exchange transitions), which is surprising if these birds respond to and repeat their calls with the same stereotyped intervals. It is suggested that the rarity of overlaps could be attributed to the short duration of calls. However, this finding is relevant to their claims that birds don't anticipate the calls of others and should be investigated further. The authors should compare the experimentally observed instances of overlap to overlap rates that would be expected by chance (based on shuffled or simulated data with the same call rates). If short call durations can indeed explain low instances of call overlaps, this may suggest that Java sparrows simply lack the need for anticipatory mechanisms seen in other species with longer calls at higher risk of call overlap/signal masking. If overlaps occur less than expected by chance, other possible explanations are auditory suppression of repeated calls by responsive calls (e.g. Larter & Ryan 2024; Norton et al 2022) or the general self-suppression of repeat calls, which could indeed be indicative of an expectation of a vocal response or the result of differential usage of repeated calls across contexts. With the current dataset, the authors could at least compare the relative frequencies of repeated calls (short component) of subject individuals in the F2F vs Pre & Post phases.

As per your suggestion, we have included the number of overlaps in pseudo turn-taking within the manuscript. However, since the number of overlaps in pseudo turn-taking is also extremely low, we interpret the scarcity of overlaps in Java sparrows as being due to their short call duration. If Java sparrows possessed a mechanism for avoiding overlap, similar to that of zebra finches, we believe that overlap would not occur even with an increased number of individuals. Taking this into account, we have revised the manuscript as follows:

L.408-414

However, in Java sparrows, the call duration is so brief that even if the birds anticipate a response, there may be no need to avoid overlapping. This can be further clarified in future experiments using larger groups. Specifically, in vocal exchanges involving more than two individuals, the likelihood of an overlap should increase as the number of interacting individuals increases,

regardless of the call duration. However, if birds do not overlap, as observed in zebra finches [37-40], the incidence of overlap does not increase with the number of individuals.

3) In each experimental pairing, the subject individual was given a 15 minute acclimation period (PRE phase), whereas the facing bird was introduced immediately prior to the test. Relocating birds has been shown to affect vocalization rates (Zai et al 2025). Did transporting birds have effects on call rates? Specifically, were there any asymmetries in calling behavior when birds were in the subject vs. facing role/more vs. less acclimated?

There was a tendency that the facing birds gave less calls than the subject birds, which was not significant. Although you have taken the trouble to point this out, we have decided not to include this analysis in the revised manuscript, because the results we reported here were only about the subject birds and not the facing birds.

Additional Minor Comments:

R2-4

Lines 40-41, 46-47: There is evidence that the inter-speaker intervals during conversational turn-taking can be influenced by linguistic information/the speech planning network (e.g. Castellucci et al 2022).

We agree with your comment that our use of “linguistic information” in the original manuscript was not precise. We do understand that the linguistic information does influence the inter-speaker intervals. But the content of the conversation is not the focus of our interest here. Therefore, we have changed the corresponding sentences as follows.

L.34-37

Notably, inter-individual intervals–the time between the end of one speaker’s utterance and the beginning of the next–exhibit a universal temporal regularity, averaging approximately 200 ms regardless of linguistic or cultural differences [2, 3].

L.38-41

As human vocal turn-taking is characterized by consistent short temporal regularity regardless of semantic content, the vocal exchange of non-human animals can be examined within the same framework. Numerous studies have explored vocal interactions across various animal species in an effort to draw parallels with human conversational turn-taking [4,5].

R2-5

Line 257: AIC should be defined as Akaike Information Criterion at first use.

We have made the corrections as suggested in your comment.

L.293-295

By comparing the Akaike information criterion (AIC), we estimated which model outperformed the null model in which the condition was removed from the fixed effects.

R2-6

Line 353: “Monotonic” rather than “Monotonous”.

We have made the corrections as suggested in your comment.

L.328-329

The distribution of the pseudo-turn-taking data was uniform and monotonic, with no clear components such as the short component observed in the exchange transition.

R2-7

Lines 382-383: It is not clear how the absence of “temporal regularity of vocal exchanges” has been determined. The authors previously defined temporal regularity as “stable time interval between vocalizations of two individuals and significant avoidance of vocal overlap” (lines 54-55). Is the “short component” distribution of exchange transitions not stable? Also, as mentioned above, the occurrence of overlaps is reported to be very low, but not assessed in terms of its significance. Greater consistency in the use of terms would be helpful here.

We completely agree with your comments that our inconsistency in the use of terms in our original manuscript is confusing. We have deleted the sentence pointed out from the revised manuscript and changed the expression as follows so that it more accurately reflects the results and clarifies the content.

L.375-377

This indicates that, unlike the vocal turn-taking observed in socially communicative mammals, including humans, Java sparrows do not expect or wait for a response from other individuals.

R2-8

*Line 401: “Why *do Java sparrows call contagiously?”*

We have made the corrections as suggested in your comment.

L.390

Why do Java sparrows make contagious calls?
