# Supplementary material for: Temporal regularities of vocal exchange in Java sparrows
Source: PLoS One. 2025 Jul 23;20(7):e0327024. doi: 10.1371/journal.pone.0327024 (PMC12286380; doi:10.1371/journal.pone.0327024)
Supplement: Attachment — Submitted filename: Reply_to_reviewer_kukuchi_kondo_koda_pone.pdf [file pone.0327024.s003.pdf]

Reply to reviewers and editors

Reviewer #1:

R1-1

The manuscript by Kikuchi et al is greatly improved following reviewer feedback. I appreciate the time the authors spent revising the manuscript to make the methodology and results clearer and the data interpretation more focused.
The generalization between birds and mammals, both of which are large and varied taxonomic groups, has been mostly eliminated. Ideally, the lingering references to the larger groups (e.g. line 25: "This contrasts with the temporal characteristics of vocal exchanges observed in mammals"), should be focused further to not suggest that the scientific community is certain that all mammals (or birds) share this trait – this is not something we can/cannot know. While the introduction does a fine job of providing more nuanced explanations of that sentence in particular, refraining from overgeneralizing should be avoided.

We are grateful for the reviewer's insightful comment. To better emphasize the point that these findings pertain to mammals studied to date, we have revised the sentence in the manuscript as follows:

> **L. 25**
> This contrasts with the temporal characteristics of vocal exchanges observed in social mammals that have been studied to date.

R1-2

There is an additional analysis that may add to the help clarify why no differences were observed between tr and te transitions. Do tr intervals change based on social context? In other words, are the tr intervals longer or shorter when the bird has no partner bird to exchange calls with? Comparing the tr interval across the 3 phases of the experiment (PRE, F2F, POST) may provide useful insight into the utility of that transition based on whether the bird is alone or not. If it is the same across all three contexts, it might indicate that the birds are not using a call to communicate with a partner birds. However, if the tr is longer in the F2F phase compared to the other two that could suggest that they are awaiting a potential response. Similarly, if the POST tr interval is different from PRE or F2F, that might also suggest the role of the call in an attempt to contact conspecifics.
This suggested analysis (or something similar) might also help address the gap that the authors identify on lines 92-95 ("…while studies on the intervals between repeated transitions remain limited"). The current manuscript, as written, focuses more on the differences (or lack thereof) between repeated and exchanged transitions. An analysis focused solely on the repeated transitions would better address this gap.

We sincerely appreciate the reviewer's insightful comments. In response to the suggestions, we have conducted additional analyses accordingly. As a result, we have added new sections to both the Methods and Results in the revised manuscript. Since no difference was found in repeated transitions based on the presence or absence of a partner, we have refrained from discussing this point in depth. We hope this approach is understandable and satisfactory to the reviewer.

> **L. 278-289**
> We further investigated whether the presence of a conspecific influenced the temporal structure of intervals in repeated transitions. Specifically, we examined whether the intervals between pre- and post-transition phases differed, using GMM estimation and a permutation test. The results showed that the distributions of transition intervals in the PRE and POST phases were remarkably similar. The estimated means (± variance) of the short-latency component from the GMM were –0.75 ± 0.01 and –0.074 ± 0.334 for the PRE and POST phases, respectively. The 95th percentile range for the difference in means, obtained via permutation testing, ranged from –1.802 to 0.489 and included zero, indicating no significant difference between the two phases. Based on this result, we combined the PRE and POST phases into a single category, hereafter referred to as the "solo" phase. We then compared the interval distributions between the solo and face-to-face (F2F) phases using the same GMM estimation and permutation testing approach to assess whether they differed.

and also

> **L. 334-341**
> When comparing the distribution of repeated transition of 'solo' and F2F phase by GMM, the estimated means (± variance) of the short-latency component were -0.75 ± 0.009 and -0.669 ± 0.035, respectively. The 95th percentile range for the difference in means, obtained via permutation testing, ranged from -0.329 to 0.505. These values include zero, indicating no significant difference between the two phases. These results suggest that Java sparrows showed vocal responses to facing individuals, as reflected in the shorter intervals during exchange transitions. However, the duration of repeated transitions was not affected by the mere presence of the other bird.

R1-3

To further strengthen the argument presented in the introduction, I encourage the authors to revisit the paragraphs on pages 5-6 (lines 96-121). I wonder if swapping the order of those two paragraphs might be helpful, particularly because lines 116-117 ("Specifically, mammals are more likely to expect and anticipate responses, whereas birds may not share the same expectations") beg a "why?" even though it has been addressed to some extent in the previous paragraph. (Also, moving this sentence might be helpful to contextualize which birds and mammals have been studied to avoid the overgeneralization to all birds and/or mammals.

We sincerely appreciate the reviewer's insightful comments. In accordance with the suggestion, we have rearranged the paragraphs and revised the expressions to improve the logical flow of the argument. We

have also carefully modified the phrasing to avoid the unintended impression that “mammals” refers to all mammalian species. The corresponding revisions have been made in the manuscript as follows.

L. 97-127

When considering the role of vocal turn-taking in animal societies, the socio-ecological structure of the species in question should be taken into account. Social mammals form stable groups with fixed membership even outside the breeding season, often including unrelated individuals. In such groups, vocal turn-taking helps maintain group cohesion by enabling individuals to signal their location and avoid separation through reciprocal vocal responses [42]. Birds, on the other hand, also form social flocks, but these tend to be more fluid and less stable than mammalian social structures. Within bird flocks, the most salient social units are often pair-mates, family members, or kin. For example, species such as Greylag geese (*Anser anser*) form matrilineal alliances [43], yet they do not necessarily maintain exclusive territories with fixed group membership, as is often observed in mammal groups. Given these socio-ecological differences, along with the fact that birds in general are capable of traveling much faster than mammals due to their ability to fly, temporary spatial separation from flock members likely poses a lower risk for birds than for group-forming social mammals. Accordingly, vocal turn-taking with an expectation of a response appears to be less essential for birds than for social mammals as a mechanism for maintaining proximity and group cohesion.

As is evident from recent zebra finch studies, however, the vocal exchange of calls in birds has been increasingly examined using a framework similar to that applied to primates. Previous studies have shown that humans, non-human primates, mammals, and birds share common vocal exchange features—specifically, short inter-individual intervals and longer intra-individual intervals, both of which help to avoid call overlap. Due to these shared characteristics, it has been hypothesized that vocal exchange may serve comparable functions across taxa. Nevertheless, as noted above, fundamental socioecological differences between social mammals and birds may shape their communicative strategies in distinct ways. Importantly, most avian vocal exchange research has focused on zebra finches, a key model species in songbird studies. These studies, which involved controlled face-to-face experiments, demonstrated significant avoidance of vocal overlap and active adjustments in call timing [37–40]. The authors of these studies proposed that such timing adjustments, which depend on the calls of others, are analogous to primate-like vocal turn-taking. Moreover, call timing was found to reflect social relationships such as pair bonding [40], suggesting that ecological functions of call timing may be more similar across taxa than previously assumed. However, these studies have mainly focused on inter-individual call intervals, and research on intra-individual repeated transitions remains limited (see [41]).

R1-4

Given that I had difficulty interpreting Table 1/Figure 3 initially, the inclusion of the antilogarithmic values is extremely helpful. Please include the x-axis legend on Figure 3, specifying that you are plotting the antilogarithmic values. Additionally, I wonder if reorganizing Table 1 might be helpful by having the columns reflect the short and long components with the mean ± variance in the cells. Then you could more easily present the log transformed and anti-log values for each component.

We appreciate the reviewer's insightful comments. We have changed the legend of Figure 3 as follows.

> **Fig 3. Histogram and estimated probability density function curve (curve line) fitted by the two modal GMM for the exchange transition (A) and for repeated transitions (B).**
> The dashed vertical lines represent the positions of the estimated mean parameter values in the short components of the GMMs. Note that although the data are log-transformed, the x-axis shows antilogarithmic values in seconds.

Aslo, we have re-arranged the Table1 as per the reviewer's suggestion. In doing so, we realized that variances should not be presented using antilogarithmic values. Therefore, we have removed the antilogarithmic variance values (previously shown in parentheses) from the table.

Transition	Data size (N)	Components	Mean	Variance	Mixture ratio
Exchange	1710	short	-0.728 (0.187)	0.023	0.322
		long	0.321 (2.094)	0.502	0.678
Repeated	3350	short	-0.669 (0.214)	0.035	0.167
		long	0.578 (3.784)	0.296	0.833

Overall, modifications to the manuscript have helped showcase how calling behavior in java sparrows differs from those of other birds/mammals to better explain the utility of contagious calling and the value of comparative studies to evaluate how and why different groups of animals generate and modify vocal turn-taking.

R1-5

Minor suggestions/observations:
1) Line 53, clarify 'it'

We have replaced 'it' by 'the caller'.

R1-6

2) Line 111-112, embed/modify the 'birds fly much faster than mammals' – there aren't many mammalian species that fly, so referring to their movement as 'travel' may be more appropriate.

We have made the corrections as per the reviewer's suggestion.

R1-7

3) Consider combining the sentences on lines 125-127 for readability.

We have changed the expression as per the reviewer's suggestion.

> **L. 132-133**
> There are also short monosyllabic calls, presumed to function as contact calls [44], and we focus on the exchange of these calls.

R1-8

4) Line 178 – replace 'enhance' with 'promote' or something similar

We appreciate the reviewer's suggestion and replaced 'enhance' by 'encourage' and modified the sentence as follows.

> **L. 182-184**
> The purpose of the PRE phase was to habituate the participants to the experimental space and encourage spontaneous vocalizations throughout the experimental session while reducing their stress.

R1-9

5) Line 179 – replace 'incubated' – "The duration of the F2F phase was 15 minutes."

We sincerely appreciate the reviewer's kindness in pointing out the typographical errors. Following the suggestions, I have corrected them accordingly.

R1-10

6) Line 181-2: replace with "During the POST phase, facing individuals were removed from the soundproof room…"

We again sincerely appreciate the reviewer's kindness. We have corrected the sentence by the suggested phrases in the revised manuscript.

> **L. 186-187**
> During the POST phase, facing individuals were removed from the soundproof room with only the preceding individuals remaining, as was the case during the PRE phase.

R1-11

7) Line 390 – use of the phrase, "affected by the vocal behavior of other individuals" doesn't seem appropriate given that if you'd found differences between the transitions you would have been able to write this exact phrase. Consider clarifying.

We appreciate the reviewer's comment. To improve clarity, we have removed the sentence and revised the preceding text accordingly.

> **L. 410-415**
> In other words, Java sparrows vocally communicated with each other without predicting or anticipating a response from the facing individual, but calling contagiously in response to others' vocalizations. This is quite different from human conversation, in which turn-taking occurs at an appropriate time by predicting the vocalizations of the person to whom he or she is speaking [1].

R1-12

8) Line 402 – modify to "To more appropriately test our hypothesis we will need to investigate vocal exchange …"

We modified this to rephrase it, along with the comment from Reviewer 2 below.

> **L. 427-430**
> To test our hypothesis that contagious calling facilitates the coordination of flock movement, vocal exchange and behavioral synchronization among multiple individuals should be investigated in more natural, flock-like settings.

Reviewer #2:

The authors have sufficiently addressed my previous comments and the revised manuscript is considerably improved.
I would just like to point out the following minor phrasing issues, which can be easily edited prior to publication:

R2-1

Lines 368-369: "Instead, they called in synchrony…" can be too easily misinterpreted as "called simultaneously", which they apparently do not do in this paradigm, given the low instances of overlap. This new sentence can be removed.

We appreciate the reviewer's suggestion and have removed the sentence accordingly. However, since we would like to retain the concept of "contagious," we have revised the preceding sentence as follows.

> **L. 392-394**
> Rather, it is likely that Java sparrows responded contagiously to the calls of others, although they did not appear to anticipate a 'response' call.

R2-2

Lines 402-404 "To test our hypothesis, we had to investigate the vocal exchanges and behavioral synchronization among more than three individuals in more natural, flock-like settings."
- This was not done here. Perhaps authors meant "we would have to investigate…".
-Also "our hypothesis" is a bit confusing here, given that the previous sentence refers to "our study". Text should specify "To test our hypothesis regarding the synchronization of flocks, we would have to investigate…" (or similar).

We completely agree with the reviewer's comment. To clarify the meaning of "our hypothesis," we have revised the sentence as follows:

> **L. 427-430**
> To test our hypothesis that contagious calling facilitates the coordination of flock movement, vocal exchange and behavioral synchronization among multiple individuals should be investigated in more natural, flock-like settings.

R2-3
Lines 414-415: It is not clear to me that the ability to avoid overlapping with a specific partner or in a playback experiment would necessarily mean that call overlaps would not increase in the presence of more callers. This new sentence can be removed.

We appreciate Reviewer 2's valuable suggestion to remove the sentence. However, since the anticipation of responses and overlap avoidance are important aspects of vocal exchanges, we have chosen to revise and retain the sentence as follows. We hope Reviewer 2 will understand our intention with this clarification.

> **L. 436-444**
> Specifically, in vocal exchanges involving more than two individuals, the chance of call overlap would be expected to increase simply due to the greater number of potential responders. If birds only respond reflexively without anticipating the timing of others' calls, such overlap is likely to occur more frequently. However, if individuals are capable of anticipating responses and coordinating their vocal timing, they may be able to avoid overlaps even in larger groups. Indeed, such overlap avoidance has been reported in zebra finches [37–40], where individuals adjust their timing to avoid calling simultaneously. Whether similar mechanisms operate in Java sparrows in more complex settings, such as those involving larger flocks, remains an open question.
